# Supplementary material for: Association of Androgen Deprivation Therapy with Osteoporotic Fracture in Patients with Prostate Cancer with Low Tumor Burden Using a Retrospective Population-Based Propensity-Score-Matched Cohort
Source: Cancers (Basel). 2023 May 18;15(10):2822. doi: 10.3390/cancers15102822 (PMC10216187; doi:10.3390/cancers15102822)
Supplement: Supplementary file 1 [file cancers-15-02822-s001.zip › cancers-2272182-supplementary.pdf]

Supplementary Table S1. General and clinical characteristics of prostate cancer patients according to androgen deprivation therapy (N=17,766)

Variables	Total (N=17,766)		Non-user (N=8883)		ADT user (N=8883)		*p-value*
	N	(%)	N	(%)	N	(%)	
Age (years, Mean (SD))	68.64	(6.79)	68.56	(6.59)	68.71	(6.99)	0.154
Year at diagnosis							
2008~2010	5854	32.95%	2683	30.20%	3171	35.70%	<0.001
2011~2013	5716	32.17%	3056	34.40%	2660	29.94%	
2014~2016	6169	34.88%	3144	35.39%	3052	34.36%	
Urbanicity							
Urban (Metropolitan areas)	12,284	69.14%	6231	70.15%	6053	68.14%	0.038
Rural (Other areas)	5482	30.86%	2652	29.85%	2830	31.86%	
Insurance type							
Self-employed insured	5359	30.16%	2773	31.22%	2586	29.11%	<0.001
Employee insured	11,788	66.35%	5913	66.57%	5875	66.14%	
Medical-aid beneficiary	619	3.48%	197	2.22%	422	4.75%	
Income level (quintile)							
Q1	2079	11.70%	921	10.37%	1158	13.04%	<0.001
Q2	1711	9.63%	793	8.93%	918	10.33%	
Q3	2089	11.76%	1001	11.27%	1088	12.25%	
Q4	3480	19.59%	1700	19.14%	1780	20.04%	
Q5	7530	42.38%	4152	46.74%	3378	38.03%	
Unknown or Medical-aid	877	4.94%	316	3.56%	561	6.32%	
Underlying disease							
Myocardial infection	1215	6.84%	555	6.25%	660	7.43%	0.002
Congestive heart failure	3521	19.82%	1578	17.76%	1943	21.87%	<0.001
Peripheral vascular disease	8252	46.45%	4080	45.93%	4172	46.97%	0.1664
Cerebrovascular disease	6864	38.64%	3295	37.09%	3569	40.18%	<0.001
Dementia	948	5.34%	443	4.99%	505	5.69%	0.0385
Chronic pulmonary disease	14,091	79.31%	7086	79.77%	7005	78.86%	0.1335
Peptic ulcer disease	13,628	76.71%	6912	77.81%	6716	75.61%	<0.001
Mild Liver Disease	12,392	69.75%	6196	69.75%	6196	69.75%	N/A
Diabetes without chronic complication	10,812	61.01%	5216	58.93%	5596	63.07%	<0.001
Diabetes with chronic complication	4697	26.50%	2235	25.25%	2462	27.75%	<0.001

Hemiplegia or paraplegia	1066	6.01%	391	4.42%	675	7.61%	<0.001
Renal disease	1740	9.82%	790	8.93%	950	10.71%	<0.001
Moderate or severe liver disease	507	2.86%	208	2.35%	299	3.37%	<0.001
AIDS	15	0.08%	6	0.07%	9	0.10%	0.4411
modified CCI							
0-1	1103	6.21%	630	7.09%	473	5.32%	<0.001
2	1658	9.33%	814	9.16%	844	9.50%	
3	1720	9.68%	941	10.59%	779	8.77%	
4+	13,285	74.78%	6498	73.15%	6787	76.40%	
SEER summarized stage							
Localized	11,560	65.07%	5640	63.49%	5920	66.64%	<0.001
Regional	2963	34.93%	3243	36.51%	2963	33.36%	
BMD test (Yes)	2142	12.06%	841	9.47%	1,301	14.65%	<0.001
Osteoporosis medications (Yes)	181	1.02%	67	0.75%	114	1.28%	<0.001
Primary Treatment							
Radiotherapy (Yes)	2717	15.29%	681	7.67%	2036	22.92%	<0.001
Surgery (Yes)	6140	34.56%	3660	41.20%	2480	27.92%	<0.001
Active surveillance	4721	26.57%	4721	53.15%	0	0.00%	<0.001
Osteoporotic Fracture (Event)	685	3.86%	220	2.48%	465	5.23%	<0.001
Death (after primary fracture in fractured group)	113	16.50%	30	13.64%	83	17.85%	0.1654
Index date ~ Death (Month, Mean (SD))	90.06	27.72	88.28	22.52	90.71	29.48	0.6837
Fracture ~ Death (Month, Mean (SD))	47.73	25.54	41.03	21.16	50.15	26.65	0.0937
Overall Follow-up Period (Month, Mean (SD))	47.71	29.90	48.57	30.45	46.86	29.31	<0.001
Follow-up Period until Primary Fracture (Month, Mean (SD))	37.62	34.00	41.99	25.12	35.55	31.28	<0.001

Supplementary Table S2. Mean group differences before and after the Propensity score matching information

Tx group	N	Mean difference (before PSM)	Matched	Mean difference (after PSM)
Non-ADT group	16,777	0.1485	8,883	0.0001
ADT group	11,694		8,883	

Supplementary Table S3. Univariate and Multivariate Cox proportional hazard ratio model for risk of osteoporotic fracture according to the sites

Variables	Hip fracture						Spine fracture						Wrist fracture					
	Univariate Model			Multivariate Model (2)			Univariate Model			Multivariate Model (2)			Univariate Model			Multivariate Model (2)		
	HR	(95% CI)	p-value	HR	(95% CI)	p-value	HR	(95% CI)	p-value	HR	(95% CI)	p-value	HR	(95% CI)	p-value	HR	(95% CI)	p-value
Androgen deprivation therapy																		
Non-user	1.000	(ref)	-	1.000	(ref)	-	1.000	(ref)		1.000	(ref)		1.000	(ref)		1.000	(ref)	
ADT user	2.397	(1.715-3.350)	<0.001	1.842	(1.298-2.615)	0.001	2.446	(1.934-3.095)	<0.001	2.080	(1.628-2.658)	<0.001	1.842	(1.401-2.422)	<0.001	1.749	(1.316-2.324)	0.000
Age at index date	1.093	(1.093-1.152)	<0.001	1.097	(1.066-1.128)	<0.001	1.089	(1.089-1.13)	<0.001	1.095	(1.074-1.117)	<0.001	0.989	(0.989-1.03)	0.361	1.003	(0.982-1.024)	0.775
Year at diagnosis																		
2008~2010	1.000	(ref)	-	1.000	(ref)	-	1.000	(ref)		1.000	(ref)		1.000	(ref)		1.000	(ref)	
2011~2013	0.840	(0.585-1.206)	0.344	1.029	(0.712-1.487)	0.879	0.692	(0.528-0.907)	0.008	0.841	(0.639-1.107)	0.217	0.892	(0.651-1.221)	0.475	0.957	(0.697-1.315)	0.788
2014~2016	0.778	(0.422-1.434)	0.421	0.789	(0.429-1.451)	0.445	1.181	(0.807-1.73)	0.392	1.202	(0.82-1.762)	0.345	0.934	(0.579-1.505)	0.779	0.960	(0.595-1.548)	0.866
Urbanicity																		
Urban (Metropolitan areas)	0.963	(0.693-1.339)	0.824	1.057	(0.758-1.474)	0.743	0.827	(0.66-1.036)	0.098	0.913	(0.728-1.146)	0.435	1.147	(0.857-1.535)	0.356	1.194	(0.89-1.6)	0.237
Rural (Other areas)	1.000	(ref)	-	1.000	(ref)	-	1.000	(ref)		1.000	(ref)		1.000	(ref)		1.000	(ref)	
Income level (quintile)																		
Q1	1.000	(ref)	-	1.000	(ref)	-	1.000	(ref)		1.000	(ref)		1.000	(ref)		1.000	(ref)	
Q2	0.596	(0.328-1.083)	0.090	0.634	(0.301-1.334)	0.229	0.966	(0.578-1.616)	0.896	1.015	(0.608-1.694)	0.955	1.072	(0.597-1.926)	0.816	1.116	(0.623-2)	0.712
Q3	0.916	(0.497-1.691)	0.780	0.904	(0.491-1.666)	0.746	1.351	(0.864-2.113)	0.187	1.351	(0.864-2.112)	0.187	1.367	(0.811-2.306)	0.240	1.397	(0.829-2.354)	0.209
Q4	0.596	(0.328-1.083)	0.090	0.571	(0.314-1.036)	0.065	1.054	(0.69-1.609)	0.809	1.003	(0.657-1.532)	0.988	1.000	(0.605-1.650)	0.999	1.026	(0.621-1.693)	0.921

Q5	0.833	(0.513-1.355)	0.463	0.722	(0.443-1.178)	0.193	1.016	(0.695-1.485)	0.935	0.903	(0.616-1.322)	0.599	0.875	(0.556-1.376)	0.563	0.913	(0.579-1.437)	0.693
Unknown or Medical-aid	1.829	(0.975-3.432)	0.060	1.126	(0.593-2.138)	0.717	1.819	(1.099-3.01)	0.020	1.147	(0.689-1.91)	0.597	1.428	(0.759-2.688)	0.270	1.314	(0.697-2.478)	0.398
SEER summarized stage																		
Localized	1.000	(ref)	-	1.000	(ref)	-	1.000	(ref)		1.000	(ref)		1.000	(ref)		1.000	(ref)	
Regional	0.484	(0.329-0.712)	0.000	0.728	(0.488-1.086)	0.120	0.725	(0.569-0.925)	0.010	1.032	(0.801-1.331)	0.805	0.906	(0.683-1.202)	0.494	0.961	(0.715-1.292)	0.793
modified CCI																		
0-1	1.000	(ref)	-	1.000	(ref)	-	1.000	(ref)		1.000	(ref)		1.000	(ref)		1.000	(ref)	
2	0.868	(0.245-3.075)	0.826	0.639	(0.19-2.146)	0.469	1.881	(0.613-5.769)	0.269	1.31	(0.449-3.824)	0.622	0.697	(0.213-2.284)	0.551	0.659	(0.211-2.064)	0.474
3	0.810	(0.228-2.869)	0.744	0.639	(0.19-2.143)	0.468	2.708	(0.926-7.923)	0.069	2.096	(0.752-5.836)	0.157	2.404	(0.91-6.348)	0.077	2.220	(0.869-5.667)	0.095
4+	2.436	(0.902-6.578)	0.079	1.384	(0.535-3.581)	0.503	4.897	(1.826-13.135)	0.002	2.841	(1.114-7.248)	0.029	2.549	(1.049-6.195)	0.039	2.218	(0.942-5.222)	0.068
BMD test (Yes)	2.591	(1.819-3.691)	<.0001	2.160	(1.497-3.117)	<.0001	2.313	(1.79-2.988)	<.0001	1.797	(1.372-2.352)	<.0001	1.281	(0.881-1.862)	0.195	1.228	(0.84-1.797)	0.290
Osteoporosis medications (Yes)	2.505	(0.929-6.758)	0.070	1.235	(0.468-3.26)	0.671	4.124	(2.368-7.181)	<.0001	2.180	(1.229-3.866)	0.008	0.219	(0.219-3.54)	0.857	0.853	(0.24-3.039)	0.807
Radiotherapy (Yes)	1.022	(0.657-1.588)	0.925	0.963	(0.615-1.506)	0.868	0.877	(0.632-1.217)	0.433	0.823	(0.591-1.148)	0.252	1.156	(0.806-1.659)	0.431	1.035	(0.714-1.499)	0.858

Supplementary Table S4. Cumulative survival rate for osteoporotic fracture in prostate cancer patients along with follow-up period

Androgen deprivation therapy	Follow-up period(year)								p-value for long-rank test
	1 year		3 year		5 year		7 year		
	No of events / No of at risk	Cumulative survival rate (95% CI)	No of events / No of at risk	Cumulative survival rate (95% CI)	No of events / No of at risk	Cumulative survival rate (95% CI)	No of events / No of at risk	Cumulative survival rate (95% CI)	
Osteoporotic fracture(Composite)									
Non-user(N=8,883)	29/7549	0.996(0.995-0.998)	70/5482	0.986(0.983-0.989)	66/3304	0.970(0.966-0.975)	44/1411	0.952(0.945-0.959)	<0.001
ADT user(N=8,883)	72/7826	0.991(0.990-0.993)	190/4991	0.962(0.958-0.967)	120/3032	0.934(0.928-0.941)	68/1312	0.906(0.897-0.915)	
Osteoporotic fracture(Subtype:Hip)									
Non-user(N=8,883)	3/7573	1.000(0.999-1.000)	17/5542	0.997(0.996-0.998)	19/3371	0.993(0.990-0.995)	9/1456	0.989(0.985-0.992)	<0.001
ADT user(N=8,883)	16/7881	0.998(0.997-0.999)	45/5125	0.991(0.989-0.993)	29/3188	0.984(0.981-0.988)	18/1411	0.977(0.972-0.982)	
Osteoporotic fracture(Subtype:Spine)									
Non-user(N=8,883)	13/7564	0.998(0.997-0.999)	31/5525	0.993(0.991-0.995)	31/3351	0.986(0.983-0.989)	17/1443	0.979(0.974-0.984)	<0.001
ADT user(N=8,883)	34/7861	0.996(0.995-0.997)	88/5083	0.983(0.979-0.986)	57/3138	0.969(0.964-0.974)	43/1370	0.951(0.944-0.958)	
Osteoporotic fracture(Subtype:Wrist)									
Non-user(N=8,883)	12/7561	0.998(0.997-0.999)	21/5531	0.995(0.993-0.997)	18/3362	0.991(0.988-0.993)	19/1451	0.983(0.979-0.987)	<0.001
ADT user(N=8,883)	22/7871	0.997(0.996-0.998)	56/5094	0.989(0.986-0.991)	39/3149	0.979(0.975-0.983)	20/1384	0.971(0.969-0.976)	
Osteoporotic fracture(Subtype:Humerus)									
Non-user(N=16,794)	1/7576	1.000(1.000-1.000)	4/5552	0.999(0.999-1.000)	2/3387	0.999(0.998-1.000)	0/1465	0.999(0.998-1.000)	0.328
ADT user(N=11,696)	1/7894	1.000(1.000-1.000)	6/5154	0.999(0.998-1.000)	3/3219	0.998(0.997-0.999)	1/1426	0.998(0.996-0.999)	

(Abbreviations) CI, Confidence Interval; Androgen deprivation therapy, ADT
